# Presidential Symposium: Compelling Evidence to Advance Gerontology Education on LGBTQIA+ Aging

**DOI:** 10.1093/geroni/igaf122.1786

**Published:** 2025-12-31

**Authors:** Laura Donorfio, Brian Chapman

## Abstract

The goal of the 2025 AGHE Presidential Symposium is to enhance awareness, foster interdisciplinary dialogue, and promote evidence-based strategies to support aging sexual and gender minority (SGM) populations within gerontology and geriatric education, practice, and research. This symposium will provide innovative approaches, pedagogical strategies, applied research, and evidence-based tools to advance inclusive curricula, policies, and services. These efforts aim to address the unique challenges faced by LGBTQIA+ older adults and to prepare future professionals to meet their needs. The first presentation shares work focused on using the film Gen Silent, as an education tool to increase aging providers knowledge and empathy for LGBTQIA+ older adults. The second presentation showcases semi-structured interview data from transgender, non-binary, and intersex (TNBI) older adults to enrich gerontology and geriatrics education by incorporating real-world narratives. The third presentation examines the unique challenges faced by older adults living with HIV in medical settings and how these experiences can be effectively incorporated into gerontology curricula through medical rhetoric frameworks. The fourth presentation explores the development of the LGBTQ Aging Toolkit and the HIV & Aging Toolkit and how they can provide ready-to-use interprofessional modules to support faculty in integrating LGBTQIA+ aging content into their curricula. We conclude with a lively discussion exploring how to support aging SGM populations by taking action through education across GSA member groups.

